# Flies spring a surprise

**DOI:** 10.7554/eLife.47720

**Published:** 2019-05-21

**Authors:** Johanna M Kobler, Ilona C Grunwald Kadow

**Affiliations:** 1 School of Life SciencesTechnical University of MunichMunichGermany; 2 Graduate School of Systemic Neuroscience (GSN)Ludwig-Maximilians-UniversityMunichGermany

**Keywords:** olfaction, innate behavior, lateral horn, neuroanatomy, cell type, *D. melanogaster*

## Abstract

A combination of genetic, anatomical and physiological techniques has revealed that the lateral horn, a region of the brain involved in olfaction in flies, has many more types of neurons than expected.

**Related research article** Dolan M-J, Frechter S, Bates AS, Dan C, Huoviala P, Roberts RJV, Schlegel P, Dhawan S, Tabano R, Dionne H, Christoforou C, Close K, Sutcliffe B, Giuliani B, Feng L, Costa M, Ihrke G, Meissner GW, Bock DD, Aso Y, Rubin GM, Jefferis GSXE. 2019. A synthetic peptide that prevents cAMP regulation in mammalian hyperpolarization-activated cyclic nucleotide-gated (HCN) channels. *eLife* **8**:e43079. doi: 10.7554/eLife.43079

**Related research article** Frechter S, Bates AS, Tootoonian S, Dolan M-J, Manton JD, Jamasb A, Kohl J, Bock DD, Jefferis GSXE. 2019. Functional and anatomical specificity in a higher olfactory centre. *eLife* **8**:e44590. doi: 10.7554/eLife.44590

When we sense an odor, our response depends by and large on two higher olfactory centers in the brain. In both mammals and insects, the innate responses to odors (such as an aversion to the smell of rotten fish) are thought to be mediated by a neural pathway in which the synapses between neurons are hardwired, whereas adaptive responses such as learning to respond to a given odor are mediated by a pathway in which the synapses are random. In the fruit fly *Drosophila melanogaster* we know a great deal about the anatomy and function of the mushroom body, the olfactory center associated with learning and memory (Aso et al., 2014a; Aso et al., 2014b; Heisenberg, 2003), but the lateral horn – the center associated with innate responses – has, in comparison, remained a black box.

Now, in two massive papers in eLife, Gregory Jefferis, Michael-John Dolan, Shahar Frechter and colleagues at the Janelia Research Campus, Cambridge University and the MRC Laboratory of Molecular Biology open this black box and provide the most profound study of the lateral horn to date (Frechter et al., 2019; Dolan et al., 2019). Besides presenting unmatched genetic tools to target its neurons, one of their most surprising findings is that the lateral horn of a fruit fly contains more neuron types than some higher brain regions in mice (Tasic et al., 2018).

In the first paper Frechter et al. report a detailed investigation of the neuroanatomical and functional organization of the lateral horn (Figure 1). They started by generating the tools they needed to reliably access the cells of this brain center. To do this they took advantage of the great number of transgenic lines that are available for flies. Put simply, these transgenes allow the expression of any gene, including a visual marker such as the green fluorescent protein (GFP), in any cell type in the brain. However, the cell type in which they drive gene expression is frequently not known, so it is necessary to proceed by trial and error. By visually screening the brains of a large number of transgenic flies under the microscope, the researchers were able to identify several transgenic lines in which GFP was expressed in neurons in the lateral horn.

**Figure 1.**
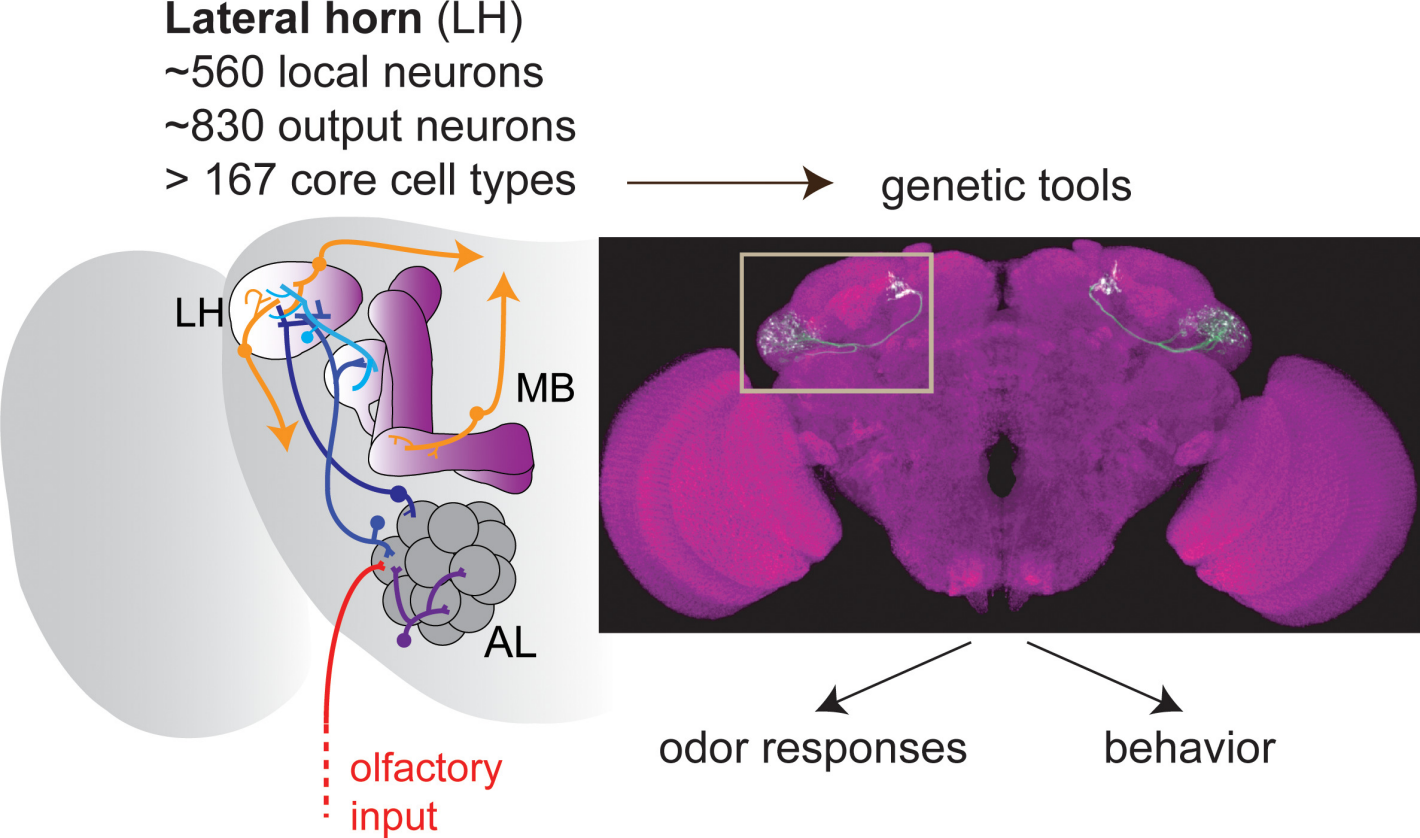
The olfactory pathway in *Drosophila*. Olfactory sensory neurons (red) provide olfactory input to the glomeruli of the antennal lobe (AL), where information is processed by local neurons before being transferred by projection neurons (blue) to the mushroom body (MB) and the lateral horn (LH). Frechter et al. combined whole-cell electrophysiology and anatomy to characterize the many different types of neurons in the LH. Dolan et al. used a range of tools to explore these neurons further. The image shows a so-called split-GAL4 line labeling a specific type of LH output neuron. Together, the two papers show that, rather surprisingly, the LH in *Drosophila* contains more neuron types than have been reported for higher brain regions in mammals.

By combining two lines with partly overlapping expression, Frechter, Dolan and colleagues created highly specific lines, called split-GAL4 lines, that mark distinct neuron types, thus allowing them to study the anatomy of the lateral horn in unprecedented detail and extent. With the help of electron microscopy (Zheng et al., 2018), they estimated that the lateral horn contains approximately 1400 neurons, which far exceeded expectations. Using a hierarchical naming system based on anatomical parameters, the researchers then clustered these neurons into 261 different types, which was also much more than expected. Some 34 of these types were neurons that were completely confined to the lateral horn (so-called LH local neurons), and 227 projected to other regions of the brain (LH output neurons).

After laying down these neuroanatomical foundations, Shahar Frechter painstakingly recorded the electrical activity of more than 500 individual lateral horn neurons in response to a broad odor set. In line with the lateral horn having a role in innate behavior, similar lateral horn neurons in different flies responded to odors in similar ways. This allowed Frechter et al. to define so-called functional cell types to describe neurons with similar morphologies and odor responses. By analyzing when neurons in the lateral horn fired in response to odor, they further showed that the output neurons of the lateral horn were significantly better in distinguishing odors by certain categories, such as distinctive chemical features, than other populations of olfactory neurons, such as the projection neurons that carry the olfactory signals into the lateral horn (Figure 1; see also Jeanne et al., 2018).

In the second paper, Dolan et al. report how the lateral horn is connected to other brain regions and start to test its role in behavior. To this end, they screened 2444 split-GAL4 lines and found that 382 of these lines targeted small subsets of lateral horn neurons. By matching these lines to the anatomical data gathered by Frechter et al., they were able to target 82 different types of lateral horn neurons.

These new genetic tools then allowed Dolan et al. to combine light and electron microscopy to create nothing short of the first atlas of a large number of lateral horn neurons. They categorized the cell-types according to their polarity, which describes whether a neuron is a local, an output neuron, or an input neuron relaying information into the lateral horn. Interestingly, they found that in addition to receiving olfactory input, the lateral horn also receives input from visual, mechanosensory and gustatory brain centers. Moreover, it sends projections to a wide variety of brain regions, but not directly to the ventral nerve cord, which suggests that olfactory information is subject to another layer of processing before any motor outputs are generated. However, one of the most exciting results was that the lateral horn receives input from the mushroom body and, moreover, that around 30% of the output neurons from the lateral horn interact with mushroom body neurons: this confirms the existence of ‘horizontal’ connections between these two higher olfactory centers for the mediation of innate and adaptive responses.

Why does the lateral horn have so many neuron types, and how does this relate to its function? To test the relationship between neuron type and behavior, Dolan et al. added optogenetics to their repertoire of experimental techniques (Figure 1). They found that activation of just two neuron types resulted in the flies demonstrating aversion behavior, and activation of just one type led to attraction. These numbers are surprisingly low, given that 6 out of 20 output neurons in the mushroom body drive such valence decisions (Aso et al., 2014b; Owald and Waddell, 2015). In line with this, several cell types in the lateral horn changed motor behaviors when activated: this suggests that an important function of the lateral horn is to instruct different types of innate behavior, dependent on odor category but irrespective of valence.

The advances reported in these two papers mean that researchers now have the tools needed to potentially understand the lateral horn at the same level of detail as we currently understand the mushroom body (Aso et al., 2014a; Aso et al., 2014b). This is important because the parallel processing seen in flies (that is, different pathways being responsible for innate and adaptive responses) appears to be a highly conserved feature of olfactory processing (and other forms of sensory processing) in vertebrates including mammals. The extensive direct and indirect connections between the lateral horn and the mushroom body also suggest that the lateral horn can modulate the output of the mushroom body, and vice versa, with such modulation ultimately having an impact on behavior.
